# A model based on intensity of medical care may improve outcomes for internal medicine patients in Italy

**DOI:** 10.1371/journal.pone.0211548

**Published:** 2019-01-31

**Authors:** Emanuele Torri, Marta Rigoni, Stefania Dorigoni, Dimitri Peterlana, Susanna Cozzio, Giandomenico Nollo, Walter Spagnolli

**Affiliations:** 1 Autonomous Province of Trento, Dipartimento Salute e Politiche Sociali, Trento, Italy; 2 Fondazione Bruno Kessler, Healthcare Research and Innovation Program – HTA Unit, Trento, Italy; 3 Azienda Provinciale per i Servizi Sanitari, Ospedale “S. Chiara” Trento, U.O. Medicina Interna, Trento, Italy; 4 Università degli Studi di Trento, Dipartimento di Ingegneria Industriale – BIOtech Labs, Trento, Italy; Istituto Di Ricerche Farmacologiche Mario Negri, ITALY

## Abstract

**Background:**

In medical wards, to guarantee safe, sustainable and effective treatments to heterogeneous and complex patients, care should be graduated into different levels of clinical intensity based on a standardised assessment of acute-illness severity. To support this assumption, we conducted a prospective observational study on all unselected admissions of 3,381 patients to a medium size internal Italian Medicine Unit by comparing Standard Medical Care model (SMC) to a new paradigm of patient admission based on Intensity of Medical Care (IMC).

**Methods:**

The SMC operated during 2013, while an IMC organizational model started in 2014. In SMC, patient’s admission was performed according to bed availability only. In IMC, after the stratification of clinical instability performed using the National Early Warning Score (NEWS) and clinical judgment, patients were allocated to three different ward areas (high, middle, and post-acute medical care). We compared clinical and organizational outcomes of IMC patients (2015) to SMC patients (2013), performing adjusted logistic regression model.

**Results:**

We managed 1,609 and 1,772 patients using SMC and IMC, respectively. The IMC seemed to be associated to a lower risk of clinical worsening for patients. Comparing IMC to SMC, the odds ratio (aOR) for urgent transfers to intensive care units was 0.69 (p = 0.03), and for combination of urgent transfers and early deaths was 0.68 (p<0.01).

**Conclusions:**

Redesigning the configuration of internal medicine ward to support urgency and competency of the clinical response by applying IMC paradigm based on the NEWS, improved outcomes in patients with acute illness and enhanced ward performances.

## Introduction

Medical wards, which lay at the heart of inpatient care, are experiencing an increase in workload and number of admissions of complex, frail and critically ill patients, commonly with undefined diagnosis and varying critical conditions [1]. All over Europe and worldwide, acute medical wards are facing the challenge of conjugating optimal levels of safety, effectiveness and patient-centeredness of care with better efficiency in the use of the limited resources available. Furthermore, it was reported that, more than any other health care setting, general medicine wards generate the errors leading to preventable deaths [2]. Factors leading to in-hospital adverse outcomes frequently include poor clinical monitoring, inadequate interpretation of changes in physiological parameters, and failure to undertake appropriate action [3, 4].

The identification of patients at risk of clinical deterioration during hospital stay and adequacy and timely of treatment is critical to deliver safe and effective acute care. Intensive care admissions may be preventable in 21% of cases and sub-optimal care may be responsible until one-third of hospital deaths [5, 6]. Understanding how to better organize care for general medical patients implementing evidence based interventions, such as those aimed at redesign and standardization of clinical processes and early recognition and treatment of the deteriorating patient, is an international priority [7].

To face this challenge in general medical wards, it seems crucial to go beyond traditional organizational models based on undifferentiated settings of care and enable a safe and appropriate management of patient flow across the continuum of treatment. An “average” standard of care delivered to all patients may indeed lead to unmet or mismatched needs, especially for critically ill patients vulnerable to clinical deterioration.

To do that could be profitable the introduction of a new paradigm of “intensity of medical care” (IMC). This promising model of medical care relies on the classification of patient acuity conducted upon admission using tools for stratification of severity of illness and the delivery of varying intensity (high, medium, low) of clinical care (i.e. staffing levels, monitoring technologies, operational procedures) in integrated medical ward areas [8]. This approach relies on the classification of patients’ acuity performed using validated tools, such as the National Early Warning Score (NEWS), originally developed to recognize impending clinical deterioration during hospital stay and provide a trigger for escalation of clinical care [9, 10].

Currently, the use of the NEWS as the recommended early warning scoring system has been approved and endorsed to standardise the approach to detecting and grading the severity of acute illness. Uptake of the NEWS has been brisk and widespread, extending from the NHS of the United Kingdom to international hospitals in Europe and beyond [11].

The NEWS provides the basis for a unified and systematic approach to the first assessment and triage of acutely ill patients, and a simple track-and-trigger system for monitoring clinical progress for all patients in hospitals [11].

Therefore, as objective stratification tool coupled with subjective physician risk assessment, it can be used to cluster patients assigned to internal medicine ward partitions by illness severity in order to deliver a graduated response, according to clinical instability and complexity of needs [12].

However, there is a paucity of data in literature on evaluating the efficiency of models based on IMC and the NEWS itself in improving clinical outcomes and organizational performances within the context of internal medicine wards.

As changes were planned in the internal medicine ward of Santa Chiara Hospital of Trento (Italy), we considered such an opportunity to design a prospective study to evaluate a new organisational model based on IMC, which replaced the traditional standard of medical care (SMC). The new IMC model merged systematic assessment of severity using the NEWS scoring system with appropriate and graduated clinical response to heterogeneity and complexity of patients, supported by technological and practical changes to promote teamwork, collaboration and performance feedback.

The purpose of our study was to examine the impact of IMC when compared to SMC on key clinical outcomes and performance measures.

## Methods

### Study design

This was a prospective observational study of patients consecutively admitted to the Internal Medicine Unit (IMU) of the Santa Chiara Hospital of Trento from January 1^st^ to December 31^st^ 2013, and from January 1^st^ to December 31^st^ 2015.

### Patients

Santa Chiara Hospital is a general public hospital with over 600 beds, providing all medical and surgical specialities. It operates as a hub of the acute hospital network in the Italian Province of Trento, which includes an additional six spoke acute hospitals. All patients admitted to the IMU from January 1^st^ to December 31^st^ 2013 were included in the SMC period and those admitted to the IMU from January 1^st^ to December 31^st^ 2015 were included in the IMC period. Although the IMC model was implemented from January 1^st^ 2014, we did not include patients admitted in 2014 as it was considered a “switching year”; we assumed that the impact post implementation would only be measurable among those enrolled at least a year following implementation. Patients were admitted to the IMU from the Emergency Room (ER), from other acute care hospitals and from the Intensive Care Unit (ICU) in a post-critical phase. Patients treated in the ER were triaged by the staff and referred for ward hospitalization, short-term observation, and/or discharge to other supportive facilities. Around 12% of ER visits were followed by a hospital admission; the IMU accounts for about one-third of all hospital admissions from ER.

### Standard of Medical Care (SMC) model

Until the end of 2013, the IMU consisted of one section with 58 beds organized with patient assignment based on medical speciality and reflecting the general principle of the “first available bed” only. Telemetry monitored beds, excluding electrocardiography, were not available. One joint (physician and nurse) ward round was performed daily with clinical revaluations if necessary; sporadically bedside non-invasive mechanical ventilation was practised.

### Intensity of Medical Care (IMC) model

Since 2014, the IMU has been managed using IMC with 54 beds differentiated into three integrated ward areas: the “High medical care”, “Medium medical care”, and “Post-acute medical care” areas.

The “High medical care” area, equipped with eight beds and with centralised multi-parameters monitoring technology (electrocardiography, pulse oximetry, orthostatic blood pressure), allowed for the provision of “high-intensity” medical care or “intermediate” care (Intermediate Medical Care Area, IMCA). There were twice-a-day joint (physician and nurse) ward rounds and clinical revaluations if necessary and non-invasive ventilation could be performed if needed.

The “Medium medical care” area, equipped with 33 beds and with telemetric monitoring parameters (electrocardiography), allowed for the provision of “medium-intensity” medical care (Medium Medical Care Area, MMCA) and once daily joint (physician and nurse) ward round and clinical revaluations if necessary.

The “Post-acute medical care” Area (PAMCA), equipped with 13 beds, allowed for the provision of “low-intensity” medical care and once daily joint (physician and nurse) ward round and clinical revaluations if necessary. In this area, urgent admissions were not planned; this area allowed for the transfer of patients entering the post-acute phase, stepping down from higher levels of care (IMCA or MMCA), needing rehabilitation, and/or with discharge problems.

Using this organisational model, an assessment on ward admission was conducted in a common area to determine appropriate allocation of patients to beds in the IMCA or MMCA, depending on the degree of clinical instability and thus risk of deterioration.

**Fig 1:**
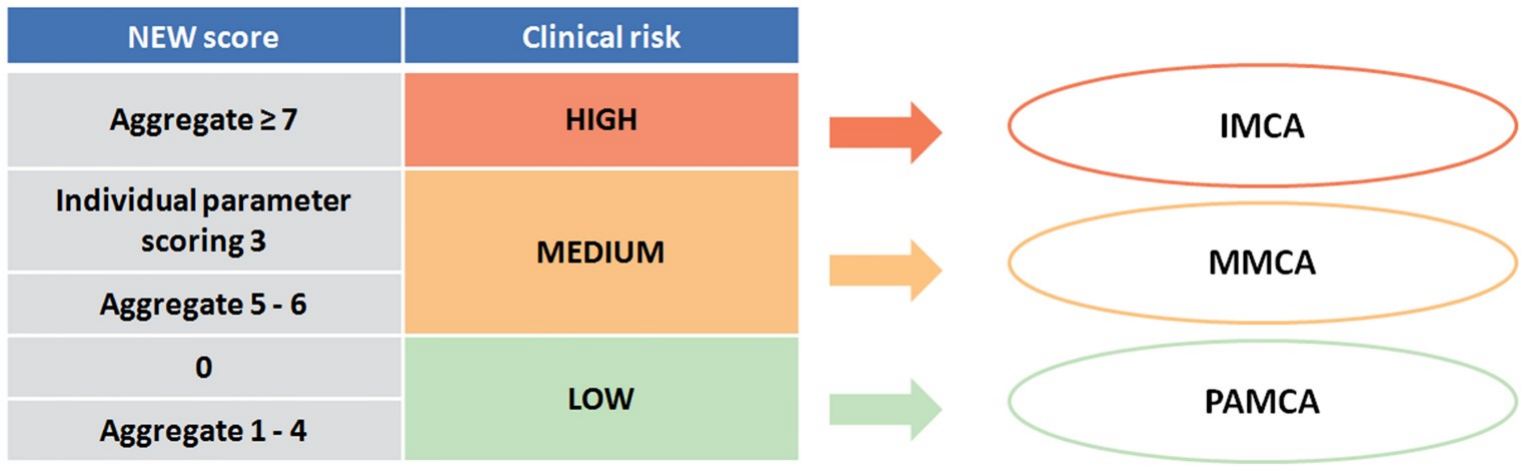
Patient placement in relation to three categories of clinical risk as assessed by NEWS [11]. At admission, patients were addressed to IMCA (Intermediate Medical Care Area) or MMCA (Medium Medical Care Area) according to individual level of risk. The Post-Acute Medical Care Area (PAMCA) served as pre-discharge area for step-down patient.

After integration in the ordinary protocol for ward admission, nursing staff trained to collect and understand the six physiological parameters routinely recorded for NEWS calculation [10] triaged all patients. According to the Royal College of Physicians [9], patients were grouped into three trigger levels/risk categories: low score (NEWS, 0–4); medium score (NEWS, 5–6); and high score (NEWS, ≥7). At admission, objective assessment based on the NEWS classification integrated by subjective physician’s evaluation of acute-illness severity served to address patient placement in the ward area with the required and most appropriate setting of care: IMCA and MMCA. Fig 1 sketches patient’s placement in relation to the three categories of risk assessed by NEWS.

### Data source, variables and outcomes

We collected data from the Hospital Information System. To protect patient privacy all data were anonymized before analyses and patient informed consent was collected. Since we did not manage sensitive data, according to the rules of the Healthcare Trust of the Autonomous Province of Trento, ethical committee approval was not needed. The following variables included in the patient discharge data were available for each patient: age, gender, date of admission, date of discharge, mortality, date of mortality, principal and secondary diagnosis (ICD-9-CM international coding system), intra-hospital and inter-hospital transfers. We considered the aggregate average relative Diagnosis Related Group (DRGs) weight to take in account the resource consumption and complexity of patient management for the two groups of patients. We classified patient morbidities into five categories: cardiovascular diseases, acute respiratory illness, diabetes mellitus, chronic kidney diseases, and neoplasia, according to the first three ICD-9-CM diagnosis.

We compared the two organizational models (SMC versus IMC) analysing the following primary outcomes:

- early in-hospital mortality (within 72 hours of admission);
- total in-hospital mortality;
- urgent (unanticipated and unplanned) transfers for clinical deterioration to a higher level of care (intensive care i.e. intensive care unit, intensive coronary unit, intensive respiratory unit);
- combined outcome one: early in-hospital mortality and urgent transfers for clinical deterioration;
- combined outcome two: total mortality and urgent transfers for clinical deterioration.

In addition, we assessed the following secondary outcomes related to management of bed capacity, and patient flow:

- proportion of stepdown patients admitted in IMU transferred from a higher level of care;
- bed occupancy as percentage of beds occupied by patients in a year;
- nursing staff to patient ratio as the number of beds each nurse has to care;
- patients’ hospital length of stay (LOS).

### Statistical analyses

We presented descriptive statistics; categorical variables were expressed as frequencies and percentages, while continuous variables were expressed as means and standard deviations (SD), and medians and interquartile ranges (IQR). We compared differences in patient’s characteristics between those receiving SMC and IMC using Pearson’s chi-squared tests, U Mann-Whitney tests, and Student’s t-tests when appropriate. Outcomes were analysed performing crude and adjusted logistic regression models. For each outcome, we calculated the adjusted odds ratio (aOR) for the IMC in comparison to SMC. We adjusted for age, sex, cardiovascular diseases, acute respiratory illness, diabetes mellitus, chronic kidney diseases, and neoplasia.

A *p*-value<0.05 was considered statistically significant. We performed all analyses using Stata statistical software, version 13.0 (StataCorp, 4905 Lakeway Drive, College Station, Texas 77845 USA).

## Results

We analysed 3,381 admissions under the different organizational models. We compared 1,609 consecutive unselected admissions during the SMC period (2013), and 1,772 consecutive unselected admissions during IMC period (2015).

As summarised in Table 1, there were no significant differences in demographic and clinical data, and in the average weight of DRGs, during SMC and IMC periods. The calculation of the average DRGs weight did not show any difference between the two investigated periods. Most frequently, causes of admission were cardiovascular diseases and acute respiratory illness; these did not vary significantly based on the model of care. Over 95% of all IMU admissions were urgent medical admissions. The distributions of LOS between SMC and IMC did not show any change (SMC median LOS 10 days, IQR 6–16 days, IMC median LOS 10 days, IQR 6–17 days; p = 0.85).

**Table 1:** Demographic and clinical characteristics of patients admitted to the Internal Medicine Unit at the Santa Chiara Hospital in Trento during the two periods.

Characteristics	Year 2013 SMC, n (%)	Year 2015 IMC, n (%)	p—value
Total	1,609	1,772	
Male	870 (54)	941 (53)	0.57
Female	739 (46)	831 (47)	0.57
Median age [IQR]	73 [62–82]	73 [62–82]	0.77
Cardiovascular diseases	1055 (66)	1180 (67)	0.53
Acute respiratory illness	666 (41)	755 (43)	0.47
Diabetes mellitus	87 (5)	90 (5)	0.67
Chronic kidney diseases	152 (9)	158 (9)	0.59
Neoplasia	326 (20)	389 (22)	0.23
Average weight of DRGs (SD)	1.19 (0.77)	1.20 (0.82)	0.78

Abbreviations: IMC = Intensity of Medical care; SMC = Standard Medical Care; IQR = Interquartile range; DRGs = Diagnosis Related Groups; SD = standard deviation. Person’s chi squared, U Mann-Whitney, and Student’s t-tests were used for comparison between the two periods.

### In-hospital mortality

**Fig 2:**
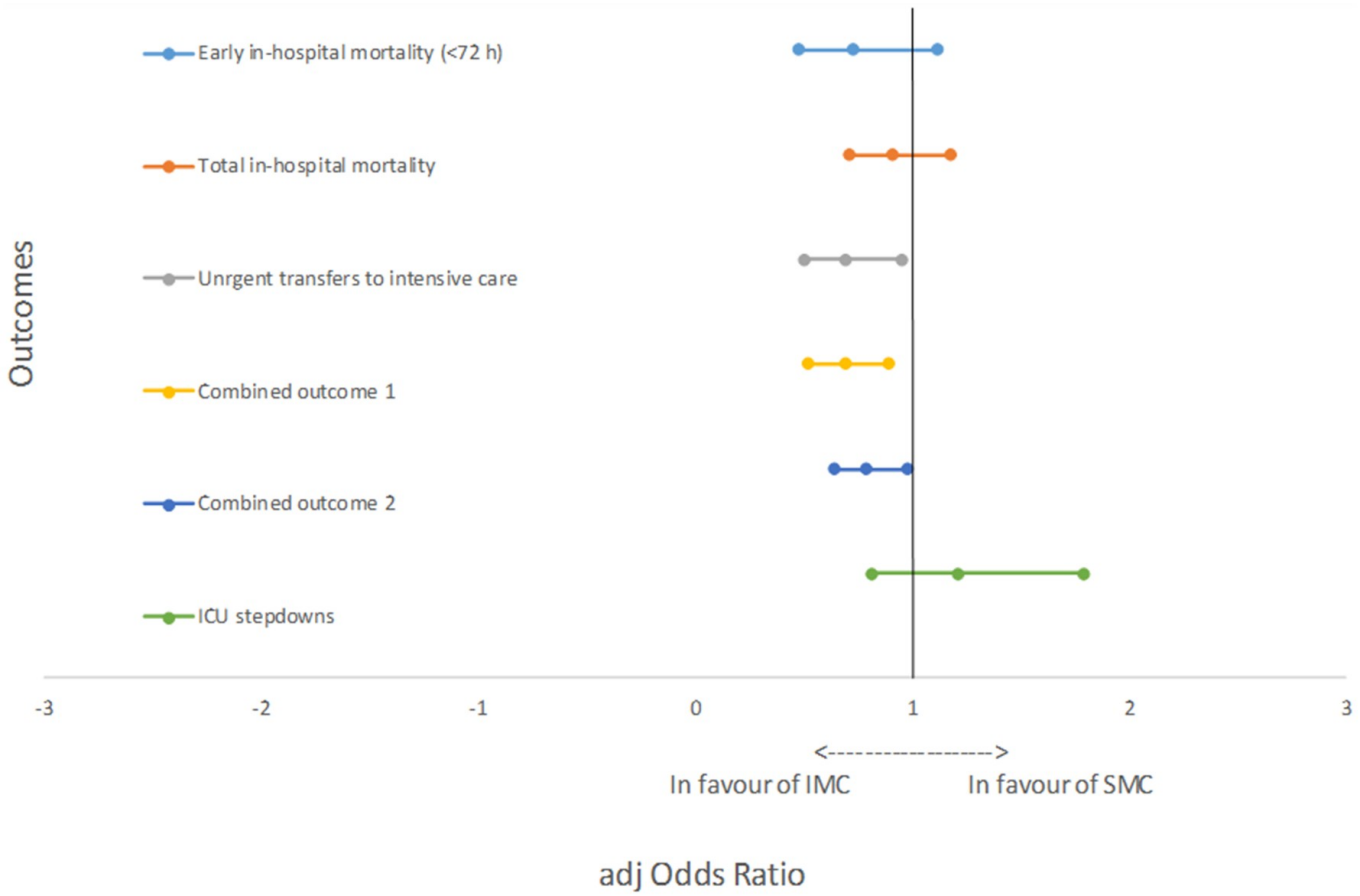
Graphical representation of the adjusted odds ratios (aOR) of the evaluated outcomes.

We did not find a statistically significant decrease in early and total in-hospital mortality from the SMC to the IMC period (Table 2, Fig 2).

**Table 2:** Studied outcomes and results comparing IMC versus SMC.

Outcome	Year 2013 SMC, n (%)	Year 2015 IMC, n (%)	aOR (95% CI)	p-value
Early in-hospital mortality (<72 hours)	48 (3.0%)	39 (2.2%)	0.73 (0.48–1.12)	0.15
Total in-hospital mortality	127 (7.9%)	129 (7.3%)	0.91 (0.71–1.18)	0.49
Urgent transfers to intensive care	87 (5.4%)	67 (3.8%)	0.69 (0.50–0.95)	0.03
Combined 1	133 (8.3%)	103 (5.8%)	0.69 (0.52–0.89)	<0.01
Combined 2	201 (12.5%)	181 (10.2%)	0.79 (0.64–0.98)	0.04
ICU step-downs	45 (2.8%)	59 (3.3%)	1.21 (0.81–1.79)	0.35
Total	1,609	1,772		

Abbreviations: combined 1 = urgent transfers for clinical deterioration and early in-hospital mortality; combined 2 = urgent transfer for deterioration and total in-hospital mortality; IMC = Intensity of Medical Care; SMC = Standard Medical Care; aOR, adjusted odds ratio; CI, confidence interval; ICU, Intensive care unit.

### Urgent transfers to intensive care

Our data showed a significant decrease in the proportion of urgent transfers to intensive care. The IMC reduced the aOR of urgent transfers to intensive care by 31% for the IMC patients when compared to SMC patients (Table 2, Fig 2).

### Combined outcomes

We found a meaningful reduction in the combined outcome one (early in-hospital mortality and urgent transfers) between the two periods. The exposition to the IMC organizational model reduced the aOR by 31% for IMC patients when compared to SMC (Table 2, Fig 2).

We found a significant decrease in combined outcome two (total in-hospital mortality and urgent transfers) between the two periods. The IMC reduced the aOR by 21% for IMC patients compared to SMC patients (Table 2, Fig 2).

### Secondary outcomes

We did not find any statistically significant changes in the proportion of step-down admissions from the SMC period to the IMC period (Table 2, Fig 2).

Bed occupancy moved from 76% to 90% from the SMC to the IMC periods. The nursing staff to patient ratio also changed from an average of 1:9 to 1:12 from the SMC to the IMC period, respectively. For the IMC model, the ratios were 1:4, 1:12, and 1:14 in the IMCA, MMCA, and PAMCA, respectively. During the study period we experienced a substantial stability in the workforce (nurses and physicians), reporting a yearly turnover rate (leavers in the year/total workforce) well below 10%.

## Discussion

This study demonstrated that in a general medical ward with a large patient population, the transition from SMC to the IMC on patient admission and care was associated with improved clinical outcomes, more appropriate bed management, and better utilization of ICU resources with no negative outcomes for patients.

The organisational change determined by IMC was associated with a decrease of patients’ condition worsening as documented by reduction in the number of unplanned transfers to intensive care, and a reduction in the combination of unplanned transfers and both early and total mortality.

The introduction of the IMC model positively acted on the efficiency of ward management as showed by the measures related to nursing/patient ratio and bed occupancy (90%), which seemed to provide a good balance between safety and care efficiency in a real life setting. According to our data, the IMC may allow for adequate levels of care for patient even with high occupation rates, which in traditional SMC models have been described as associated with higher risk of adverse events [13]. Additionally, the increase number of step-down ICU patients admitted in internal medicine ward may lead to optimization of beds management of the hospital system as a whole, particularly considering the importance of hospital beds availability in the ICU for critical patients [14, 15].

Thus, this study shows that systematic triage and stratification of patients based on risk of clinical instability triggering the most appropriate level for clinical response for ongoing acute care may improve outcomes for acutely ill patients and increase efficiency of bed management in an internal medicine ward. Our findings demonstrated the validity of embracing the NEWS to aid decision making with regard to the most appropriate clinical setting for ongoing care.

To our knowledge, there are no comparable prospective pre and post studies of models of “intensity of medical care” based on grades of acute-illness severity as defined by the NEWS implemented in internal medicine wards. Models of medical care enabling a systematic assessment of clinical acuity and appropriate and graduated clinical response to heterogeneity and complexity of patients across the continuum of care were endorsed in several countries, including Italy [16]. Implementing local policies defining pathways for safe, effective, efficient, and seamless escalation and transfer of care is an increasingly important focus of activity across Europe and worldwide [11].

However, in general, evidence of effective implementation and durability of changes proposed to improve the quality and safety of care in medical wards is limited in comparison to other acute hospital settings [2]. Furthermore, comparing results at international level may not be easy or appropriate because factors such as standardisation of terms, regional/local policies and evidence-based protocols regarding care practices or ICU admission criteria may act as confounder [17].

We think that some analogies may be found in experiences conducted in hospitals implementing intermediate care units (IMCU), structurally similar to our IMCA, born to enable a more appropriate use of beds, allowing management of those patients otherwise inappropriately and unnecessarily admitted to the Intensive Care Unit [17].

Recently, a multicentre European study showed for the first time, that adults admitted to hospitals with both Intensive Care Units (ICU) and independent Intermediate Care Units (IMCU) had lower in-hospital mortality than those admitted to ICUs without an IMCU [18].

In our study, the hospital setting for acute unstable patients was obtained with implementation of an IMCA adequately equipped for observation and intervention, inside the medical ward, similarly to the methods reported in other studies on intermediate care beds. However, the sole implementation of IMCA, which could resemble other studies on intermediate care/high dependency units, was not enough to explain our results achieved.

We implemented the IMC programme in a healthcare system pursuing a long-term comprehensive quality strategy [19]. Setting up a new model of care requires system-wide changes in practice, that in our case were implemented through strong clinical leadership commitment, alignment and integration of clinical improvement efforts with organizational priorities, systematic collaboration among physicians and nurses based on the use of a common language. It was also essential to establish an infrastructure for regular feedback on data and performances. Outcomes were chosen singularly or in combination, among those more frequently proposed in the literature, also for being useful, understandable by all clinicians and administrators, and adequate to represent clinical deterioration [20]. From the physician perspective in internal medicine, mortality and urgent transfers reflected two complementary (and “not competing”) outcomes, recognized as valid measure of relevant events linked to clinical quality of care.

Dealing with the effectiveness of early warning scores, the way in which the human element of healthcare service (e.g., physicians) perceives and reacts to the risks is very important to understand and assess healthcare quality and resource allocations [21].

Impact of change, variation of clinical practice and outcomes may be influenced by patient variables and due to complex organisational and contextual factors affecting systems of care [22, 23]. Influence of quality improvement interventions on outcomes may require time and “maturity”, reflecting the developmental stage and consolidation of efforts to secure positive change [24]. Therefore, factors other than the specific features of new organisational model of care might explain changes in outcomes achieved in our study, including mortality and admissions to the ICU.

As shown in results, the patient population treated under SMC did not show any meaningful difference from the population treated under IMC, both considering demographic, clinical data, and under DRGs (as proxy of clinical workload) and the staff was similar in the two periods.

### Study limitation and results generalizability

We realized a prospective and before-after study in a single centre in a real-life hospital setting. We performed an observational study, which was the only options once beds were reorganised and intermediate care beds set up, considering the great pressure to admit patients if capacity permits and ethical issues. Context-related constraints prevented an adequate selection of controls based on stable and comparable organisational and clinical features. Due to these organizational constraints, we were not able to conduct a controlled before-after study. This limits us to infer causality from our results since we could not determine the effect of the intervention whilst taking into account baseline trends over time [25]. We are not aware of influence of any secular trend that might explain changes or skewed outcome measures over the study period.

To reduce selection bias, we included all patients admitted during the two periods analysed.

We implemented a dynamic and multifaceted improvement intervention purposefully evolving over time in response to learning. Therefore, to avoid bias due to learning curve, a period of one year (2014) was excluded by the analysis. We decided to consider the second year of IMC implementation as fully development period.

In our study, we used outcome measures, singularly or in combination, widely proposed in studies evaluating impact of scoring systems, intermediate care models and quality improvement interventions [20, 26, 27]. We did not investigate any economic and cost related outcomes. However, we did consider performance measures that are commonly associated with an appropriate use of hospital resources.

The evidence base could be increased by further research addressing also patient-perspectives as well as staff-reported outcomes and resources utilization.

## Conclusions

In internal medicine wards, it is of utmost importance to standardize the assessment of acute-illness severity, to implement changes in order to establish graded care options between intensive and conventional care, and to support urgency and competency of the clinical response throughout the course of hospitalization.

The new organisation of internal medicine ward areas resulted in meaningful improvement of patient outcomes and efficiency of patient flow and bed management. We believe this was a valuable intervention allowing for the restructuring of the management of acute medical patients in large hospitals.

Therefore, a model framed on intensity of medical care may be associated with outcomes that are more favourable. Intensity of medical care may constitute a framework upon which other simple, innovative, structured and feasible interventions for quality and safety in the medical ward can build.

## Supporting information

S1 File (XLSX)
